# The Professional Nurse Self-Assessment Scale: Psychometric testing in Norwegian long term and home care contexts

**DOI:** 10.1186/s12912-015-0109-3

**Published:** 2015-11-16

**Authors:** Elisabeth Finnbakk, Sigrid Wangensteen, Kirsti Skovdahl, Lisbeth Fagerström

**Affiliations:** School of Health and Medical Sciences, Örebro University, Fakultetsgatan 1, Örebro, 702 81 Sweden; Lovisenberg Diaconal University College, Lovisenberggt. 15 b, Oslo, 0456 Norway; Faculty of Health, Care and Nursing, Gjövik University College, Postbox 191, Gjövik, 2802 Norway; Faculty of Health Sciences, Buskerud and Vestfold University College, Postbox 7053, Drammen, 3007 Norway; Åbo Akademi University, Vasa Campus, Postbox 311, Vasa, 65101 Finland

**Keywords:** Advanced practice nursing, Clinical competence, Factor analysis, Long term care, Professional home nursing, Psychometrics, Questionnaires, Self-assessment

## Abstract

**Background:**

Nurses’ clinical competence is vital to ensure safe and high quality care, and the continuous assessment of nurses’ clinical competence is of major concern. A validated instrument for the self-assessment of nurses’ clinical competence at different educational levels across specialties and countries is lacking. The aim of this study was to test the reliability and construct validity of the new Professional Nurse Self-Assessment Scale (ProffNurse SAS) questionnaire in long term and home care contexts in Norway. The questionnaire is based on the Nordic Advanced Practice Nursing model, in which the nurse-patient relationship is central.

**Methods:**

The study has a cross-sectional survey design. A purposive sample of 357 registered nurses who worked in long term and home care contexts in two geographical regions encompassing eight municipalities and three counties was included. The respondents completed the 74-item ProffNurse SAS questionnaire and demographic background data was collected. Data collection was conducted in two phases: first region autumn 2011 and second region spring 2012.

Exploratory factor analyses (EFA) were used to test the psychometric properties of the questionnaire and included the following steps: assessment of the factorality of the data, factor extraction by Principal Component Analysis (PCA), oblimin (oblique) factor rotation, and interpretation. Cronbach’s alpha was used to estimate the internal consistency.

**Results:**

The PCA revealed a six-component structure, reducing the number of items in the questionnaire from 74 to 51. Based on the content of the highest-loading items, the six components were named: Direct Clinical Practice, Professional Development, Ethical Decision-Making, Clinical Leadership, Cooperation and Consultation, and Critical Thinking. The Cronbach’s alpha values ranged from 0.940 (highest; Direct Clinical Practice) to 0.737 (lowest; Critical Thinking), leading to the estimation that the ProffNurse SAS is reliable.

**Conclusions:**

The six components support the study’s theoretical framework. The ProffNurse SAS showed acceptable reliability and construct validity and may therefore be a promising instrument for the assessment of practicing nurses’ clinical competence. However, we recommend further psychometric testing in other countries and contexts and the inclusion of larger samples of nurses at various levels of education, particularly master’s level APNs.

## Background

Throughout the world, health care services are undergoing continuous and rapid changes related to demands for cost effective yet safe and high quality health care. In that adverse health care events threaten the realization of high quality care in all care settings [1], it is imperative to improve patient safety [2]. The importance of nurses’ roles and competence in ensuring patient safety has been confirmed in several studies [3, 4]. Naylor et al. [5] found positive linkages between nursing and patient care quality, and nurses hold an important and leading role in improving health outcomes [6]. A global, well-educated nurse workforce is needed.

Many countries are facing shortages in the health care workforce [7, 8]. This not only relates to the ability to maintain a sufficient number of care staff but also to the ability to provide a suitable mix of nursing competence, with the aim to ensure patient safety in all care contexts. The systematic assessment of nurses’ actual competence levels in diverse care settings has therefore become ever more crucial and of interest to educators, healthcare managers, and politicians on all levels. To ensure the clinical competence of nurses, constant monitoring and supervision is needed. Nurses themselves need to be cognizant of whether their own competence levels meet the standard required for their actual roles. Systematic assessment also enables the tracking of competence throughout an individual career and the assessment of whether nurses’ competence levels meet the requirements of the health care services. Thus measuring nurses’ competence may be useful for a variety of reasons.

During the past decades, advanced practice nurse (APN) roles have been successfully established (e.g., in the USA, Canada, Great Britain, Holland, New Zealand, and Australia). The APN role includes educational advancement, specialization, and role expansion [9] and is shaped by country and context specific characteristics [10]. It is nevertheless recommended that the role be held by individuals with a master’s level degree in nursing [10–12] that also includes a concentration in an APN role [9, p. 79]. When the APN role is implemented, promising effects of the redistribution of professional roles are seen: where certain tasks, including responsibility, are transferred from physicians to APNs. The fact that APNs provide care and treatment of equal or even better quality than physicians is of interest [13, 14]. APN roles and models are emerging in the Nordic countries at the moment. Even at this early stage, it is possible to discern an emphasis on the importance of clinical competence [15, 16] and that the role transition from registered nurse (RN) to APN appears to be a maturation process that encompasses a broader and deeper holistic view of the patient’s state of health [17]. A recent Nordic study also revealed that top-level managers and politicians emphasize that the acute and complex needs of ill older people will require nurses who possess an advanced competence, relative to both medical treatment and nursing care [18].

During the last decade, interest in assessing nurses’ competence has clearly increased [cf. 19–26]. Still, Watson et al. [27] report that until 2002 the concept “competence” was poorly defined and that a lack of rigor in the instruments used for its assessment existed. We carried out a comprehensive literature search of the Medline and CINAHL databases using the following key terms: research/assessment/measurement combined with clinical competence/nursing competence/advanced clinical competence/advanced clinical practice, and instrument/tool/scale. The literature search was augmented by an examination of the collected articles’ reference lists. The search process resulted in several instruments for nurses’ self-assessment of competence.

In Jordan, the Competency Evaluation Questionnaire was developed to assess the competence levels of nursing graduates [28]. In China, the Competency Inventory for Registered Nurses, which compares nursing competency and organizational climate [29], was developed and validated as having the potential for cross-cultural application [30]. In Taiwan, three instruments were developed: the Clinical Nursing Competence Questionnaire [31], the Public Health Nurse Professional Competency Scale [32], and the Clinical Competence Questionnaire [23]. In Japan, two instruments were developed: the Holistic Nursing Competence Scale [33] and the Competence Scale for Senior Clinical Nurses [19]. To detect differences in competence across countries in Europe, the European Health Care Training and Accreditation Network developed the EHTAN Questionnaire Tool (EQT), a nurse competence self-assessment tool for general nurses [20, 34].

In Finland, the generic Nurse Competence Scale (NCS) was developed [24]. The NCS includes 73 items and is based on Benners’ domains of clinical practice [35]. The NCS has been used to assess the competence of graduating nurses [22] and to measure or compare nurse competence in different work environments [25, 36–39]. The NCS has also been translated into various languages and used across cultures and countries, for example in Lithuania [21], Iran [40–42], the USA [43], Norway [44], and Australia [45]. The NCS has furthermore been validated in an Italian study [46]. Still, even though Meretoja et al. [24] provide extensive documentation of the development of the NCS, during testing of the German version in Switzerland Müller [47] reported that the original seven-factor structure of the NCS was not confirmed. A recent psychometric test of the NCS in Norway has also concluded that the original NCS factor structure was not confirmed [48].

In Sweden, a new instrument has been developed based on formal national competence requirements: the Nurse Professional Competence (NPC) Scale [26]. The NPC was developed for use prior to graduation and among practicing registered nurses over time.

Nilsson et al. [26] maintain that only a few instruments are psychometrically sound. To date, instruments have mostly been developed for use in hospital contexts. In general, the focus of instruments has been the assessment of nurses’ clinical competence at the generalist level (basic level qualifications or a bachelor’s degree). However, when the development of new APN roles started in Finland and Sweden at the turn of the 21st century, a clear need existed for a new instrument for the self-assessment of clinical competence at different educational levels and across specialties and countries.

Wilkinson [49] maintains that without the right tools to assess competency it is difficult to know if nurses are safe to practice. To our knowledge, no instrument as yet exists that measures nurses’ clinical competence at different educational levels. Therefore, the aim of this study was to test the reliability and construct validity of the new Professional Nurse Self-Assessment Scale (ProffNurse SAS) questionnaire in long term and home care contexts in Norway.

### Theoretical framework

The epistemological foundation of the ProffNurse SAS is grounded on a life learning perspective and covered by the three Aristotelian dimensions of knowledge: *epistêmê*, *technê,* and *phronêsis*. Epistêmê represents nurses’ theoretical scientific knowledge, technê the knowledge in doing, and phronêsis practical wisdom [50, 51]. The theoretical framework of the ProffNurse SAS is based on the Nordic APN model, which is a modified version of the International Council of Nurses’ (ICN) and Hamric’s definitions of the central competence domains of advanced nursing practice [9, 11]. The ICN defines an APN as a registered nurse who has acquired the expert knowledge base, complex decision-making skills and clinical competencies for expanded practice [12]. Hamric moreover emphasizes that while many of the same interventions are performed in basic and advanced nursing practice, advanced nursing practice is based on deeper and broader competencies [9].

In the Nordic APN model it is assumed that competence domains remain the same on the generalist, specialist, and advanced levels. This is supported in a recent study finding that clinical competence is deepened but not actually changed between levels [17]. A holistic approach and a central nursing science perspective, including health, ethos, and caring as the core of nursing, are emphasized. The model comprises eight core competencies: direct clinical practice, ethical decision-making, coaching and guidance, consultation, cooperation, case management, research and development, and leadership [52, 53]. The focal point of these competencies is the dynamic and mutual nurse-patient relationship, where truly “knowing the patient” is the core of clinical competence. In the model the concept “clinical competence” encompasses the synthesis of epistêmê, technê, and phronêsis [50, 51] and can therefore be described as “knowledge in actions”.

## Methods

### Design

The study has a cross-sectional survey design and constitutes the first phase of psychometric testing of the new ProffNurse SAS instrument. The study sample included RNs at the generalist and specialist levels, with some having completed master’s level studies in nursing. Still, as the master’s level studies were not concentrated to an APN role, these RNs were not APNs.

### Instrument development

The first version was originally named the Nurse Clinical Competence Scale (NCCS) and was developed in the Swedish language in Finland in 2004 [54]. While the NCS provided the inspiration, with the NCCS the researchers sought to strengthen the assessment of clinical skills on an advanced level, including variables such as history taking, physical assessment, and clinical decision-making.

Translation of the Swedish-language NCCS for the purposes of this study into the target language Norwegian was guided by the nine-step procedure of Wild et al. [55]. A five-person committee oversaw the translation, including forward and backward translation [55]. The second and third authors (SW, KS) conducted the forward translation from Swedish into Norwegian. The first, second, and third authors (EF, SW, KS) conducted the reconciliation. An external, independent translator with no prior knowledge of the instrument performed the back translation. All authors participated in the review of the back translation and final harmonization. A second external, independent, bilingual translator with no prior knowledge of the instrument then translated the Norwegian version into English. The last phase of the translation process included research group discussions about the reconciliation, back translations, and final harmonization.

The Swedish-language NCCS consisted of 67 items. After an assessment and revision of these 67 items, the research group decided to add seven new items to strengthen the patient perspective and supplement medical and skills aspects. The name of the instrument was changed to ProffNurse SAS. To assure face-validity, five independent experts assessed the clarity, wording, understanding, and relevance of the questionnaire. Fifteen RNs from four nursing homes reviewed the questionnaire’s form and content, the time needed for completion, and the clarity of the accompanying information letter. These groups deemed only a small number of revisions necessary. A 10-point Numeric Rating Scale (NRS) was used in the ProffNurse SAS. The numeric options for the NRS were enclosed in ten boxes and the scale ranged from 0 to 5 at 0.5 intervals; zero indicated a lack of competence while 5 indicated full competence. Response options are equidistant and therefore provide interval level data [56]. Respondents were asked to tick the box representing the numeric option best describing the quality of their performance related to each of the items.

### Sample and data collection

RNs working in long term and home care contexts in eight different municipalities in three Norwegian counties and from all educational levels were included in the study. Municipalities represented both rural areas and small to midsized urban areas (30–60.000 inhabitants). The eligible number of practicing RNs was 704. While all of them were invited to participate in the study, only 371 questionnaires were returned (response rate 52.7 %). Fourteen questionnaires were rejected as incomplete with 26 items not answered (>35 %), resulting in 357 complete surveys. No APNs were among the eligible population. Even though master’s level programs in APN are offered in Norway, such a level of studies is relatively new and the APN role is still emerging. Demographic background data were collected.

Among other variables, a reliable factor analysis depends on sample size. Determining sample size is challenging because of various “rules of thumb”: e.g., 100 participants as a minimum required [56, p. 513] or ratios of participants to items such as 5 to 1 or 10 to 1 [57, p. 190]. While Field [58] emphasizes that experts differ on what the cases-to-variables ratio should be, he ultimately suggests that it makes little difference to the stability of factor solutions and recommends that the sample size be “300 or more” [58, p. 684]. Following Field’s recommendations, we found that the 357 respondents who returned complete questionnaires constituted an acceptable sample size, with the ratio 4.8 per item.

Questionnaires were delivered in envelopes marked with the name of the first author, together with an information letter. To ensure respondent anonymity the questionnaires were marked with code numbers indicating the respective nursing homes or home care departments in the particular geographic regions. The questionnaires were completed anonymously, returned in sealed envelopes, and delivered to boxes or shelves centrally placed in reception offices.

Data collection took part in two phases: the first region (covering two counties) during September - November 2011 and the second region (covering one county) during April–June 2012. In the first region there were 16 independent units (11 nursing homes and 5 large organized home care departments), while in the second region there were 19 independent units (9 nursing homes and 10 home care departments). The head nurses of the participating units acted as contact persons and administered questionnaires and reminders to all potential respondents. Two reminders were sent: the first about 14 days after the initial start of the study, the second about 2 weeks after this first 14-day period. The number of participating RNs from each unit ranged from 2–35.

### Data analysis

The PASW Statistics for Windows, Version 18.0 was used for analyses. Exploratory factor analyses (EFAs) were used to test the psychometric properties of the questionnaire. Principal Component Analysis (PCA) was used as the method for extraction for all EFAs. The EFAs were conducted as follows: assessment of the factorality of the data, factor extraction, factor rotation, and interpretation. The intention was to reduce the number of items [59] and avoid duplication of questions while still retaining meaningful factors. To test the level of correlation between items (i.e. internal consistency) Cronbach’s alpha tests were performed.

The Kaiser-Meyer-Olkin (KMO) measure of sampling adequacy was performed to test partial correlation between variables. KMO values range between 0 to 1, and a KMO value in the 0.8–0.89 range is considered “meritorious” [58, p. 685]. An absolute value of 0.4 is recommended as the cut-off value for factor loadings [60]. However, the research group chose 0.3 to determine whether items with factor loadings close to 0.4 should be included due to emerging theoretical considerations.

The first EFA was carried out with extraction based on eigenvalues >1, which yielded 18 components. With this method the number of components is often overestimated [59, 61, 62]. Parallel Analysis is reported to be the most accurate method of determining the number of components to be extracted [56, 57, 62]. When using the Monte Carlo PA software program to perform Parallel Analysis [57], the program asks for the number of items, the number of respondents in the actual study, and how many replications are desired. Average eigenvalues from the random data sets are calculated and compared to the eigenvalues of components (from the initial EFA). Only components with greater eigenvalues than the average from the random data sets are retained [57]. Parallel Analysis was performed with 100 replications as recommended [57, p. 200] and resulted in 6 components to be extracted.

The second and third EFAs were performed with the 6 components defined for extraction. In the second EFA, all 74 items in the ProffNurse SAS were included. The third EFA was performed with the 53 items remaining after the analysis of the second EFA. The rotation method, oblimin (oblique) rotation with Kaiser Normalization, was chosen because the possibility of correlations between the components being extracted existed. When oblique rotation is performed both structure matrix and pattern matrix are reported, which gives a different picture than when the components are correlated [57]. These structure and pattern matrices indicated that the components were correlated. The component correlation matrix (not shown in table) supported this result, showing multiple correlations > .3 [57]. Hence, oblique rotation was a suitable choice.

### Ethical considerations

The study has been conducted in accordance with The Declaration of Helsinki [63] and the Ethical Guidelines for Nursing Research in the Nordic Countries. The study has been reviewed and assessed by the Norwegian Social Science Data Services 2011 (ref no. 26431). Access to the field was obtained from the chiefs of the included municipalities. The return of a questionnaire was regarded as informed consent to participate in the study, anonymously and voluntarily.

## Results

The overall response rate (*N* = 704) was 52.7 % (*n* = 371). The response rate for the first region was 51.% (*n* = 166) and the second region 54.% (*n* = 205). Of the total sample 80.7 % were nurses in direct care (*n* = 300), 11.3 % were nurse managers (*n* = 42), and 1.6 % were administrative nurses (*n* = 6). 95.4 % were women (*n* = 354). The respondents’ mean age was 41.5 years (range 22–68), with average working experience 9.8 years (range 0–32). Of the total sample 40.2 % (*n* = 149) possessed education above the generalist level, with 2.9 % (*n* = 11) educated on the master’s level.

In the second EFA 49.2 % of the variance was explained, and there were 680 (25 %) non-redundant residuals with absolute values >0.05. The Structure Matrix (Table 1) shows correlations between variables and components before rotation. Table 1 demonstrates that 28 items had loadings ≥0.4 to one component, 20 items to two components, 17 items to three components, and 3 items to four components. Six items had no loading exceeding 0.4 to any of the components.

**Table 1:** ProffNurse SAS (*n* = 357)—Structure Matrix

	Component					
Item	1	2	3	4	5	6
17	**.784**	.206	.384	.396	.172	−.331
27	**.751**	.258	.308	.256	.170	−.320
18	**.721**	.085	**.403**	.281	.112	−.318
34	**.710**	.147	**.409**	.292	.239	**−.425**
23	**.704**	.329	.363	.218	−.053	−.369
25	**.702**	.361	.189	.069	.292	−.147
31	**.701**	**.429**	.389	.238	.112	−.287
24	**.693**	.212	.173	.089	.208	−.196
30	**.681**	.207	**.416**	.370	.090	**−.541**
36	**.679**	**.470**	**.426**	.112	.299	−.294
19	**.677**	−.013	**.523**	.396	.156	**−.499**
20	**.656**	.005	**.448**	.333	.116	−.388
28	**.646**	**.452**	**.470**	.217	.064	**−.435**
35	**.630**	.035	.286	.242	.218	**−.456**
16	**.625**	.156	.232	.399	.242	−.368
26	**.623**	.298	**.474**	.235	.227	**−.442**
22	**.597**	.297	**.519**	.224	.142	**−.416**
29	**.579**	**.542**	**.440**	.140	.088	−.363
38	**.560**	.364	**.471**	.065	.258	−.235
37	**.548**	**.442**	**.490**	.066	.361	−.291
21	**.530**	.257	**.439**	**.473**	.127	**−.501**
55	**.453**	.383	**.414**	.196	**.453**	−.397
10	.397	.392	.267	.376	.161	−.080
32	.183	.069	.127	.151	.009	−.130
50	.260	**.699**	.332	.362	.254	**−.453**
52	.327	**.698**	.266	.233	**.433**	−.172
11	.314	**.688**	.270	.351	.285	−.153
63	.151	**.673**	.254	.252	.377	−.326
51	.326	**.626**	**.415**	.387	.190	**−.500**
59	.308	**.621**	.344	.276	.168	−.388
49	.232	**.607**	.201	.200	.140	−.119
62	.344	**.591**	.302	.283	.111	**−.431**
66	.323	**.553**	.222	.294	**.404**	**−.403**
70	**.400**	**.533**	**.400**	.318	.054	**−.420**
14	.237	**.489**	.184	.230	.091	−.109
58	.264	**.441**	**.415**	.140	.151	−.388
64	.168	**.440**	.348	.134	.179	−.201
69	.135	.340	.215	−.026	.112	−.164
65	.195	.337	.300	.219	.147	−.251
44	.384	.332	**.768**	.202	.073	−.320
40	.399	.242	**.764**	.332	.302	**−.482**
42	**.485**	.089	**.760**	.284	.164	−.363
43	**.483**	.164	**.760**	.188	.077	−.335
41	.361	.142	**.755**	.366	−.049	−.341
45	.365	**.454**	**.748**	.092	.244	−.313
39	**.428**	.290	**.742**	.349	.268	**−.466**
7	.278	.372	**.626**	**.449**	−.167	−.241
48	**.424**	.384	**.625**	.372	.117	**−.549**
46	.301	.141	**.622**	.355	.199	**−.453**
54	.192	.346	**.606**	.372	.010	−.196
53	.152	.398	**.533**	.350	.213	−.271
74	.268	.339	**.504**	**.495**	.145	**−.447**
47	.070	.087	.225	.151	.025	−.155
3	.220	.253	.260	**.678**	.252	−.231
2	.294	.239	.213	**.644**	.267	−.188
5	.324	.278	.189	**.634**	.321	−.124
1	.155	.174	.365	**.607**	.013	−.324
8	.340	.227	**.468**	**.569**	.024	−.377
6	.198	.348	**.471**	**.563**	−.120	−.240
9	.335	.194	**.428**	**.549**	.233	−.240
15	.301	**.405**	**.436**	**.520**	.029	−.255
12	.313	**.444**	**.437**	**.455**	.144	−.335
4	.058	.124	.174	.361	−.173	−.159
72	.113	.199	.152	.130	**.774**	−.071
71	.151	.238	.151	.169	**.718**	−.161
67	.344	.310	.113	.329	**.469**	−.292
60	.302	.326	.367	.140	.082	**−.792**
61	262	.300	.287	.118	.076	**−.720**
56	.389	.236	.260	.312	.336	**−.644**
73	.386	.151	.533	.357	.187	**−.641**
33	.463	.022	.332	.247	.099	**−.620**
57	.360	.258	.365	.196	**.513**	**−.590**
68	**.513**	.391	**.444**	.343	.091	**−.572**
13	**.528**	.079	.392	.502	.210	**−.531**

Extraction method: Principal Component Analysis

Rotation method: Oblimin (oblique) with Kaiser Normalization

Loadings ≥0.4 in bold

The Pattern Matrix (Table 2) shows the unique contribution of a variable to a component. Two items (items 29 and 62) have loadings ≥ 0.4 to more than one component, while the remaining items with loadings ≥ 0.4 only loaded to one component. Table 2 demonstrates that 19 items had loadings ≥0.4 to component one, 11 items to component two, 11 items to component three, 7 items to component four, 2 items to component five, and finally 6 items to component six (loadings ≥0.4 for all items). Two items loaded >0.4 to more than one component, while all the other components, with the exception of 18 items with no loading ≥ 0.4, loaded only to one of the components.

**Table 2:** ProffNurse SAS (*n*−357)−Pattern Matrix

	Component					
Item	1	2	3	4	5	6
24	**.731**	.067	−.096	−.076	.067	.036
25	**.722**	.229	−.087	−.114	.137	.119
17	**.719**	−.037	.053	.205	.010	.002
27	**.714**	.063	−.019	.058	.004	−.038
18	**.673**	−.153	.156	.090	−.020	−.022
23	**.654**	.177	.036	−.005	−.240	−.113
31	**.623**	.264	.081	.012	−.072	.024
34	**.597**	−.109	.121	.076	.100	−.148
36	**.562**	.288	.165	−.151	.129	.017
20	**.558**	−.261	.222	.146	.004	−.113
35	**.545**	−.195	.000	.064	.102	−.269
16	**.542**	−.055	−.109	.261	.109	−.140
30	**.534**	−.028	.059	.147	−.074	−.300
19	**.520**	−.324	.275	.184	.042	−.215
28	**.495**	.279	.157	−.051	−.124	−.162
26	**.451**	.069	.208	−.014	.079	−.172
38	**.432**	.183	.318	−.181	.130	.059
29	**.430**	**.408**	.158	−.125	−.097	−.102
22	**.426**	.078	.287	−.028	−.003	−.137
37	.370	.249	.316	−.202	.226	.002
21	.317	.027	.117	.280	−.016	−.258
10	.300	.275	.052	.273	.042	.192
32	.138	.002	.026	.098	−.037	−.047
11	.118	**.609**	−.002	.206	.135	.099
52	.119	**.608**	.014	.060	.290	.069
63	−.140	**.600**	−.002	.085	.252	−.186
50	−.052	**.599**	−.008	.163	.090	−.285
49	.092	**.584**	−.016	.072	.007	.057
59	.059	**.527**	.051	.074	.010	−.208
62	.120	**.503**	−.031	.086	**−.508**	−.274
51	.014	**.490**	.080	.167	.023	−.306
14	.125	**.455**	−.018	.126	−.027	.061
66	.079	**.431**	−.105	.122	.265	−.250
70	.179	**.412**	.096	.106	−.113	−.212
64	−.037	.355	.239	−.027	.092	−.035
58	.020	.321	.237	−.077	.035	−.231
69	.011	.306	.133	−.162	.040	−.077
65	.006	.235	.151	.085	.062	−.104
44	.107	.105	**.724**	−.074	−.028	.007
43	.255	−.102	**.721**	−.084	−.013	−.001
42	.235	−.218	**.712**	.030	.088	−.020
45	.061	.239	**.709**	−.212	.139	.008
41	.098	−.111	**.708**	.143	−.129	−.025
40	.041	−.065	**.662**	.057	.216	−.170
39	.094	−.001	**.618**	.079	.168	−.119
54	−.073	.183	**.553**	.205	−.070	.085
46	−.010	−.121	**.517**	.147	.133	−.218
7	.039	.217	**.506**	.273	−.282	.058
53	−.160	.229	**.441**	.179	.138	−.034
48	.107	.154	.385	.110	−.023	−.295
47	−.052	.009	.186	.082	−.001	−.075
3	−.005	−.077	.013	**.639**	.175	−.011
2	.121	.067	−.048	**.610**	.183	.044
5	.173	.111	−.074	**.605**	.233	.133
1	−.091	.008	.171	**.538**	−.057	−.147
6	−.037	.214	.297	**.454**	−.224	.004
9	.117	−.030	.252	**.441**	.157	.040
8	.102	.022	.238	**.433**	−.082	−.133
15	.078	.257	.215	.388	−.093	.009
4	−.050	.076	.050	.341	−.225	−.079
13	.324	−.189	.082	.338	.098	−.315
74	−.046	.138	.281	.322	.039	−.227
12	.056	.284	.197	.291	.018	−.091
72	−.084	.034	.155	.056	**.779**	.078
71	−.059	.076	.051	.080	**.700**	−.024
67	.184	.162	−.172	.229	.374	−.142
55	.216	.173	.191	−.034	.337	−.165
60	−.001	.179	.045	−.120	−.057	**−.775**
61	−.003	.179	−.019	−.108	−.051	**−.726**
56	.135	.036	−.085	.125	.219	**−.555**
33	.288	−.193	.055	.056	−.006	**−.521**
73	.077	−.109	.300	.128	.086	**−.470**
57	.061	.027	.188	−.029	.421	**−.463**
68	.280	.201	.101	.104	−.078	−.368

Extraction method: Principal Component Analysis

Rotation method: Oblimin (oblique) with Kaiser Normalization

Loadings ≥0.4 in bold

The eighteen items with loadings <0.4 were excluded from further analysis. High levels of non-response may identify problem items. Deletion may be an option when missing values on variables are not central to the analysis [60], and recommendations for item deletion range from 15 %–40 % [64]. We decided to exclude three items due to internal missing items and employed limits to determine which should be excluded. Items 49 (8.4 % missing), 71 (12.6 % missing), and 72 (17.6 % missing) were subsequently excluded. Although the chosen limits for exclusion may seem rather rigid, these three items had greater missing values than the other, included items. In total 21 items were excluded. An overview of these items and why they were excluded are shown in Table 3.

**Table 3:** Excluded items

Item	Content	Highest loading	Component	Not included due to
4	I am self-critical when it comes to my work	.341	4	Loading <0.4
10	I am a good example to others at my workplace	.300	1	Loading <0.4
12	I share my experiences with others at my workplace	.291	4	Loading <0.4
13	I apply my clinical expertise in caring for patients	.338	4	Loading <0.4
15	I encourage my colleagues	.388	4	Loading <0.4
21	I carry out an overall evaluation of the nursing care	.317	1	Loading <0.4
32	I evaluate the effect of the medical treatment	.138	1	Loading <0.4
37	I give health promotion and illness preventive recommendations in accordance with national guidelines to patients	.370	1	Loading <0.4
47	I have a supportive ongoing dialogue with patients about their needs and wishes	.186	3	Loading <0.4
48	I focus on relatives’ need for support and guidance	.385	3	Loading <0.4
49	I actively develop my own specialist area of competence (areas for further education)	.584	2	8,4 %/30 respondents did not respond to the item
55	I maintain cooperation with colleagues from the specialist health service	.337	5	Loading <0.4
58	I am familiar with my colleagues’ work tasks in relation to nursing and clinical paths	.321	2	Loading <0.4
64	I report all “near incidents”	.355	2	Loading <0.4
65	I report all incidents in accordance with the actual patient safety system	.235	2	Loading <0.4
67	I defend well-functioning routines/systems in spite of opposition from other staff	.374	5	Loading <0.4
68	I integrate theoretical knowledge into clinical practice	.368	6	Loading <0.4
69	I develop and adapt clinical guidelines based on tenable research findings and a systematic review of the literature	.306	2	Loading <0.4
71	I assess patients’ health needs by telephone	.700	5	12,6 %/45 respondents did not respond to the item
72	I give health promotion advice and recommendations to patients by telephone	.779	5	17,6 %/63 respondents did not respond to the item
74	I believe that I do a proper job	.322	4	Loading <0.4

The third and final EFA—from which the 21 items mentioned above were excluded—was then performed. In this EFA 33.9 % of the variance was explained by factor one. Furthermore factor two explained 6.6 % of the variance, factor three 5.5 %, factor four 3.8 %, factor five 3.5 %, and factor six 2.8 %. There were 353 (25 %) non-redundant residuals with absolute values >0.05. The structure matrix of this EFA (not shown in table) demonstrated that 19 items loaded >0.4 to one component, 16 items loaded to two components, 12 items to three components, and 5 items to four components. Item 14 (“*I convey the knowledge within my own specialist area to others at my workplace*”) had no loading exceeding 0.4 to any of the components.

The pattern matrix of this final EFA (not shown in table) demonstrated that no items loaded more than 0.4 to more than one component. Nineteen items loaded ≥0.4 to component one, 5 items to component two, 11 items to component three, 6 items to component four, 6 items to component five, and 2 items to component six. Two items were excluded due to low loadings in the final EFA (item 51—highest loading -.335 and item 14—highest loading -.307). Despite loadings of <0.4 two items (59 & 62) were kept in component six after discussions in the research group. Reliability tests were performed with and without each of these items, and the alpha values were higher when these items were included. The final version of the ProffNurse SAS is shown in Table 4 and consists of 51 items sorted into six components.

**Table 4:** The clinical core competencies of the Professional Nurse Self-Assessment Scale (ProffNurse SAS)

Component	Item #	Item content	Loading	Cronbach’s alpha
Direct Clinical Practice	25	I am independently responsible for health assessment (systematic physical examination), examinations and treatment of patients with complicated medical conditions	.791	0.940
	24	I am independently responsible for health assessment (systematic physical examination), examinations and treatment of patients with uncomplicated medical conditions	.766	19 items
	27	I plan and prioritize nursing and medical interventions	.706	
	17	I identify patient’s health problems	.674	
	18	I assess patient’s symptoms	.621	
	23	I evaluate and modify patients’ medical treatment	.619	
	31	I exclude differential diagnoses when assessing patients’ health conditions	.612	
	36	I interpret, analyze and reach alternative conclusions about patients’ health conditions after a detailed mapping of health history and health assessment (physical examination)	.599	
	34	I apply both subjective and objective methods when examining, treating and caring for patients	.576	
	16	I carry out systematic clinical examinations of my patients	.536	
	35	I utilize medical equipment in an appropriate and accurate manner	.529	
	28	I have knowledge of the effects of medication and treatment for the patients I am responsible for	.479	
	20	I assess the patient’s health	.477	
	30	I identify deviations in the patients' state of health and state of disease	.457	
	38	I develop and administer health-promoting and illness-preventive actions for patients	.452	
	19	I assess changes in the patient’s pathological picture	.431	
	26	I systematically gather information from each patient about his/her health resources	.428	
	29	I have knowledge of the interactions of various types of medication and what side-effects they may cause for the patients I am responsible for	.424	
	22	I take preventive actions regarding the patient’s medical problems	.412	
Professional Development	52	I generate a creative learning environment for staff at my workplace	.700	
	63	I participate in quality development work at my workplace	.675	0.830
	11	I take responsibility for competence development at my workplace	.627	
	66	I improve routines/systems that fail to meet the needs of patients at my workplace	.532	5 items
	50	I take active responsibility for my own professional development	.447	
Ethical Decision-Making	43	I take patients’ mental health needs (mood swings, feelings of hopelessness, depression, etc.) into account when assessing and planning for the health and life situation of patients	−.745	0.904
	44	I take patients’ spiritual health needs (feelings of meaninglessness, existential needs, beliefs, fear of death, etc.) into account when assessing and planning for the health and life situation of patients	−.734	11 items
	42	I take patients’ physical health needs (illness, pain, disabilities, etc.) into account when assessing and planning for the health and life situation of patients	−.731	
	41	I adopt an ethical approach in my relationship with patients	−.727	
	40	I identify and assume responsibility for patients’ own health resources in planning nursing care	−.683	
	45	I take patients’ social health needs (leisure activities, friends, financial situation, etc.) into account when assessing and planning for the health and life situation of patients	−.679	
	39	I support and guide patients in mastering their illnesses and health problems	−.644	
	54	I maintain an ethical approach towards my colleagues	−.596	
	53	I take active responsibility for creating a good working environment	−.539	
	46	I put emphasis on patients’ own wishes when assessing and planning for nursing care and medical treatment	−.519	
	7	I act ethically when caring for patients	−.485	
Clinical Leadership	5	I make my own decisions in my work	.713	
	3	I work systematically	.691	0.786
	2	I work autonomously	.676	
	1	I take full responsibility for my own actions	.558	6 items
	9	I am correct and accurate in speech and writing	.514	
	8	I understand the consequences my decisions may have for patients	.467	
Cooperation and Consultation	60	I experience a division of responsibility between the physician and me as a nurse	−.824	
	61	I cooperate well with the physician	−.783	0.820
	56	I consult other professional experts when required	−.563	
	57	I cooperate actively with other health professionals when coordinating the patient’s nursing, care and treatment	−.530	6 items
	33	I am cognizant of when my medical knowledge is insufficient when assessing patients’ health conditions	−.524	
	73	I document the steps taken in assessing patients’ needs for nursing, care and treatment	−.456	
Critical Thinking	6	I reflect on my actions	−.439	
	70	I analyze and evaluate my work continuously	−.410	0.772
	59	I perceive opportunities and have visions for how nursing and clinical paths for patients can be developed	−.357	4 items
	62	I have a vision of how nursing should be developed at my workplace	−.357	

The number of items in each component varies between 4 (lowest) and 19 (highest). The names assigned the components were derived from the content of the items with highest loadings in each component [65] and are as follows: Direct Clinical Practice (19 items), Professional Development (5 items), Ethical Decision-Making (11 items), Clinical Leadership (6 items), Cooperation and Consultation (6 items), and Critical Thinking (4 items). Factors with ≥ 5 items and factor loading >0.5 are considered “solid factors” whereas factors with <5 items are considered “unstable” [66]. With respect to internal consistency, the highest Cronbach’s alpha value was found for Direct Clinical Practice (0.940) and the lowest for Critical Thinking (0.772). Grove et al. [65] defined 0.70–0.79 as moderate Cronbach’s alpha values for newer instruments, and 0.70 is also reported as being acceptable [58]. Accordingly, the internal consistency of the ProffNurse SAS may be considered good.

## Discussion

The results of the Kaiser-Meyer-Olkin (KMO) measure of sampling adequacy showed appropriate intercorrelations for the 74 scale items to explore the underlying structure. The factorality of the data was good [cf. 57]. The PCA (Principal Component Analysis) revealed a six-component structure reducing the items from 74 to 51. Reliability is a premise for validity [65]. For this factor structure Cronbach’s alpha values ranged from 0.737–0.940, leading to the estimation that the ProffNurse SAS is reliable [67].

The first component, Direct Clinical Practice, contains 19 items with a Cronbach’s alpha of 0.940. Bland and Altman [68] describe 0.90 as a minimum and 0.95 as a desirable value for Cronbach’s alpha. Though highly correlated items may make a scale overlong with the possibility of over-emphasizing some aspects, items that are too similar may be redundant [69]. Tavakol and Dennick [67], however, recommend a maximum Cronbach’s alpha of 0.90; they maintain that Cronbach’s alpha exceeding this maximum is an indication that redundant items may be present and suggest shortening the test if such occurs.

The first component consists of 19 items, which may be considered broad. Nevertheless, all of the items in this component loaded between 0.412 (lowest) and 0.791 (highest) and only to this component. As mentioned previously, this instrument was intentionally developed to strengthen the measuring of nurses’ clinical skills. It is therefore important to include all these items in order to capture the complexity of nurses’ clinical competence at all levels, even with the risk that some of the items might be redundant. Defining and finding consensus for the concept “competence” is still under debate, but agreement seems to be emerging [70]. Competence is dynamic and relational, and according to Takase and Teraoka [33] it is a synthesis of knowledge, attitudes, values, and skills; when a holistic approach is applied, ethics and context are included [71, 72]. Clinical competence can be described as “knowledge in actions” when based on the Aristotelian view of knowledge [cf. 73]. Nurses’ clinical competence is essential to ensuring patient safety and high quality nursing care in all caring areas. Therefore this first component, Direct Clinical Practice, covers important aspects of nurses’ clinical practice at different educational levels.

The epicenter of nurses’ clinical competence is the dynamic and mutual nurse-patient relationship: the core of clinical competence is truly “knowing the patient”. The formation and fostering of such a therapeutic relationship is also the core of person-centered care [74]. In this term the word “person” involves all those who are engaged in caring and is underpinned by mutual respect, respect for others as human beings, the right to self-determination and understanding. The third dimension, Ethical Decision-Making, is built on these values and reveals that taking care of patients’ physical, social, mental, and spiritual needs is a holistic as well as a moral commitment in relation to nurses’ clinical competence.

The theoretical framework of the ProffNurse SAS supports all six components. These components encompass the eight domains seen in the Nordic APN model [52], which strengthens the validity of the instrument. To some extent, the sixth component (Critical Thinking) is relevant to the domain Research and Development seen in the Nordic APN model. To incorporate nursing knowledge into practice, critical thinking is necessary [cf. 75, 76]. We perceive critical thinking as a crucial part of nurses’ clinical competence, which implicitly includes how nurses in the Aristotelian manner integrate their scientific knowledge (epistêmê) into their practical doing (technê) in order to provide high quality and safe nursing care to the best for their patients (phronêsis) - whether in an acute care setting or not. The sixth component (Critical Thinking), therefore, corresponds to the content of the domain Research and Development seen in the Nordic APN model.

Item 48 (“*I focus on relatives’ need for support and guidance*”) had too low loading to be included. A patient’s relatives are important partners for nurses in regard to cooperation, and an investigation of this item should be included in further testing of the instrument. Items 64 and 65 (“*I report all ‘near incidents’*” and “*I report all incidents in accordance with the actual patient safety system*”) also had to be excluded because of low loadings. Item 37 (“*I give health promotion and illness preventive recommendations in accordance with national guidelines to patients*”) and item 72 (“*I give health promotion advice and recommendations to patients by telephone*”) were not included because of low loadings (see Table 3). Nevertheless, because of the importance of these items to the dimension coaching and guidance as defined in the theoretical framework, these items should be included in the next step of the development of the ProffNurse SAS. Also items 48, 64, and 65 should be evaluated for inclusion in larger samples in other contexts in further development of the instrument.

### Methodological considerations

In this study the 74-item ProffNurse SAS was tested using PCA, which is the factor analysis available in the PASW Statistics for Windows, Version 18.0. The assumption of normal distributions was not checked, as this is not required unless analyzed results are to be generalized beyond the sample [56]. Nevertheless, Sheng and Sheng [77] hold that non-normal distributions may add bias in estimating internal consistency reliability and that normally distributed variables would improve a solution [59]. A test-retest was not performed, but we recommend that during a future test of reliability this should occur. A test-retest is a stability check for how constantly respondents’ score from one occasion to another [78] and should be conducted in a short time span. Respondents’ inconsistency in answers could be due to unclear items. If so, reviewing and rephrasing should be considered.

The sixth component (Critical Thinking) was the weakest in the component structure, as two of its four items had low factor loadings. To capture the exact content, this component should be investigated in a future study.

The study sample included RNs at generalist and specialist levels and even some with some master’s level training in nursing, but in this Norwegian context there were no actual APNs. An international study is currently ongoing, in which the clinical competence of nurses at specialist level in Norway are compared to the clinical competence of APNs at master’s level in other countries.

Due to the limited sample size, the generalizability of this study should be handled with caution. The sample is not random but purposive and drawn from two strategically chosen geographic regions with the aim to capture rural and small to midsized urban areas. All RNs in these regions were invited to participate in the study.

The research group was informed that the likelihood existed that not all in the target population had e-mail access, and therefore a paper-and-pencil-based survey was chosen. The response rate was 52.7 %, and while half of the possible respondent group was reached it is possible that respondents may have been affected by selection bias. However, head nurses in both research areas informed the research group that other research projects were ongoing at the same time as our data collection. As the participation in this study was anonymous, in line with the Norwegian Social Science Data Services 2011, it was not possible to compare the demographics of the respondents to non-respondents.

When developing a new scale, the larger the starting item pool the better [78]. There is nonetheless a limit concerning what might be feasible or realistic to administer. There is also a risk of boring respondents. If the respondents considered the 74-item questionnaire to be too lengthy, this may have lowered not only the response rate but also the reliability of the received responses. In self-assessment approaches another potential response bias exists as well. The so-called Social Desirability Response (SDR) can affect validity [78, 79]. In our study, the respondents may have been motivated by potential professional expectations. To adjust for SDR it is possible to incorporate a SDR scale [78, p. 101], though to be a suitable assessment tool such a scale must be neither too brief nor too long. While we consider our final 51-item scale to be lengthy, it is still efficient, practical, and not too time-consuming and therefore appropriate. Subsequently we chose not to include a SDR scale. An effort was made to reduce the length of the questionnaire and the time estimated to complete it, while still preserving the optimal balance between brevity and reliability [59, 60, 78].

## Conclusions

The six ProffNurse SAS components (Direct Clinical Practice, Professional Development, Ethical Decision-Making, Clinical Leadership, Cooperation and Consultation, and Critical Thinking) are both reliable and valid in this actual study. The ProffNurse SAS is therefore a promising instrument. Nevertheless, we recommend further psychometric testing of the ProffNurse SAS in other countries and contexts and the inclusion of larger samples of nurses at various levels of education, particularly master’s level APNs.

## Abbreviations

APN: advanced practice nurse
EFA: exploratory factor analyses
EQT: ETHAN Questionnaire Tool
ICN: International Council of Nurses
KMO: Kaiser-Meyer-Olkin
NCCS: Nurse Clinical Competence Scale
NCS: Nurse Competence Scale
NPC: Nurse Professional Competence Scale
NRS: Numeric Rating Scale
PCA: Principal Component Analysis
ProffNurse SAS: Professional Nurse Self-Assessment Scale
RN: registered nurse
SDR: Social Desirability Response
